# Nanoethics, Science Communication, and a Fourth Model for Public Engagement

**DOI:** 10.1007/s11569-017-0302-9

**Published:** 2017-08-03

**Authors:** Andy Miah

**Affiliations:** Chair of Science Communication & Future Media, School of Environment and Life Sciences, University of Salford, Manchester, Peel Building, Room G50, The Crescent, Salford, Greater Manchester M5 4WT UK

**Keywords:** Nanotechnology, Citizen science, Scientific agency, Social media, Narrative ethics

## Abstract

This paper develops a fourth model of public engagement with science, grounded in the principle of nurturing scientific agency through participatory bioethics. It argues that social media is an effective device through which to enable such engagement, as it has the capacity to empower users and transforms audiences into co-producers of knowledge, rather than consumers of content. Social media also fosters greater engagement with the political and legal implications of science, thus promoting the value of scientific citizenship. This argument is explored by considering the case of nanoscience and nanotechnology, as an exemplar for how emerging technologies may be handled by the scientific community and science policymakers.

## Introduction

The years from 1995 to 2005 may have belonged to genetic science, as far as science communication is concerned. As the Human Genome Project neared completion, public attention was focused on how it would usher in a new era of gene therapy applications, which would attend to all kinds of health problems. Yet, it became a decade that was widely described as a failure to communicate science effectively, as stories of designer people - rather than therapeutic interventions - became the focus of public concern. Since then, greater sophistication has emerged in research into science communication, along with new proposals about how scientists should make sense of their relationship to the public.

One might expect, then, that the years between 2005 and 2015—as the decade of nanoscience—would have shown considerably more accomplished endeavours to communicate what is at stake with this new science [1], and perhaps even greater gains in public understanding as a result. Yet, this latter decade was also defined by the rise of a new form of communications—social media—the end of which was marked by the prominence of discussions about *fake news* and *alternative facts*. While a significant amount of the discourse around fake news was intimately connected to the US electoral campaign of 2016, it must be understood as the peak of a set of wider concerns that communication has become a more challenging task, because of the proliferation of digital publishing.

The evidence for this is found within an inquiry by the British Government [2], which spoke of the public’s enthusiasm in science, but also their lack of trust in scientific journalism. This loss of trust may be partly attributed to wider diminishing trust in journalism which, in the UK certainly, results from the phone hacking scandals of 2011. However, there is also a wider explanation for this loss of trust, which has applicability more globally and which has to do with how the authoritative position of traditional media has been usurped by the proliferation of other digital publishing spaces and by the transformation of our daily habits of media consumption arising from mobile connectivity.

Anxious of the consequences of such change, the British Government [2] launched an inquiry into science communication in 2016 and this article speaks to some of their conclusions. In particular, it explores how speculation and narrative are critical components of a science communication journey, while also arguing that the aspirations of upstream public engagement are best served by engaging the public on the moral and ethical dimensions of science. In doing so, it considers the opportunities that arise from relocating science communication away from traditional media structures towards the blogosphere and how this trend can be seen as an antecedent to the rise of *citizen science* and *participatory bioethics*, which have become new forms of social expectations of science. It argues that, whereas the case of communicating genetics revealed a gap between the science and the public, the case of communicating nanotechnology reveals the crisis of legitimacy in communicating science (as the British Government inquiry reveals), particularly where this relies on traditional models of media relations.

Underpinning this article is the concern that the value of science communication is often expressed exclusively in terms of its capacity to serve scientific investment, rather than to function as a means of nurturing scientifically engaged citizens. The emphasis of such work is placed often on its capacity to inspire people into science or scientific careers, as the STEM agenda attests. Yet, to achieve the kind of involvement sought by the UK’s governmental inquiry—and to achieve the kind of accumulation of ‘science capital’ that Archer et al. [3, 4] describe as crucial to building science education—it is necessary to engage people on a more critical level. Doing so in a way that resonates with how they make sense of their own lives and an individual’s sense of right and wrong is an effective means through which to do this. Foregrounding the moral implications of scientific discoveries or achievements, I argue, helps to democratize society’s pursuit of scientific discovery in a way that resonates with other societal aspirations. These conditions must be taken into account to ensure that science progresses as a project that is co-produced, collectively owned, and crucially meaningful to the people’s lives. Moreover, these principles are necessary to uphold, in order to achieve the transformative potential of communicating science and reinforce arguments made by Perrault [5].

The paper is structured in three parts. First, it examines the conditions of the debate surrounding the case of nanotechnology, drawing attention to how a moral panic about its development emerged very early on and the role of speculation in this discourse. Second, it describes insights from science communication work on nanotechnology over the last decade, which reveal how a mature and strategic investment into this work has assisted science in developing greater public understanding and engagement. Within this section, I also outline how the open-ended nature of communicating science—that there is always more that can be done—needs adjustment, focusing the public on entry points into making science meaningful to their lives, rather than simply understood. In this aspect, I build on Miah [6] by relying on the utility of presenting science as a series of ethical propositions about humanity’s future, rather than an attempt to explain scientific complexity. Finally, these developments are situated within the methodological framework of research into science communication and public engagement, from the deficit to the upstream models, through which I outline the importance of narrative ethics as a tool towards promoting public understanding. In this respect, I draw on communication principles of social media to develop a fourth model of public engagement with science, described as the *scientific agency model*.

This exposition resonates further with the British Government’s Select Committee inquiry, particularly its questions around the utility of the public debate surrounding the naming of a new research ship by the Natural Environment Research Council (NERC). When NERC launched the public poll calling for suggestions, their exercise in public engagement quickly became an ‘exercise in humour’ (Warman cited in British Government [2]) when the name ‘Boaty McBoatFace’ became the most popular suggestion, a suggestion that grew in such popularity that it became the most successful proposal. NERC claimed that the public debate around this debacle led to their becoming the most ‘well known research council in the world’ [7]. However, the incident crystallized one of the underpinning questions of science communication, which concerns whether or not it leads to public engagement, understanding, or involvement with science. In response to the suggestion that the exercise was, in fact, not a great example of public engagement via social media (presumably because the public did not demonstrate having taken seriously the task), NERC suggested that the hard work of capitalizing on such public debate would begin after its occurrence and has subsequently claimed it to have been a tremendous success. But, how does one capitalize on wide reaching, superficial awareness, and translate it into involvement with science, as a route towards greater understanding or appreciation for the value of scientific investment? This question is answered in part within this essay by examining how to nurture scientific citizenship.

The model I present does not reject the importance of the upstream approach to engaging the public early on in the process of scientific development. Indeed, it does not intend to replace previous models of pubic engagement at all, and in this respect, it may also be distinct. One of my criticisms of the three models is that they are often treated as methodologically hierarchical, when it is more useful to consider which are the best parts of each model and establish when each may be useful. Thus, while one might reject the deficit model in certain situations, there is a need to understand when it might be useful as an engagement methodology. After all, its continued use, as documented by Lee and VanDyke [8], may be more a matter of preference than of a resistance to dialogue. The scientific agency model I propose is, nevertheless, different from the other three in that it espouses a notion of public engagement that gives equal value to highly speculative science, as it does to matters of urgent debate. To elaborate on this, it is useful to look in more detail at the case of nanoscience.

## Speculations About Nanoscience

The value of speculation to science communication is hotly contested. Indeed, the current concerns about fake news, fake science, and fake research are intimately connected to the manner in which speculation about future directions in science is played out within the media, by scientists who are asked what kind of world may ensue as a result of new discoveries. Yet, the role of speculation is central to our capacity to make sense of science and this section focuses on the complexity of these factors.

Early speculation about the impact of nanoscience resembled reactions to other new scientific discoveries: opinions were often extremely polarized, ranging from claims that the new discovery or application will be a panacea for all of the society’s ills to claims that it will bring about humanity’s demise. Respectively, these views focused either on the opportunities that the technology may create or on the mayhem that it could cause. Two prominent public intellectuals demonstrate this division when commenting on nanoscience at a time when it was becoming part of a mainstream conversation about science:

in around 20 years we will have the means to reprogramme our bodies’ stone-age software so we can halt, then reverse, ageing. Then nanotechnology will let us live for ever....Ultimately, nanobots will replace blood cells and do their work thousands of times more effectively....Within 25 years we will be able to do an Olympic sprint for 15 minutes without taking a breath, or go scuba-diving for four hours without oxygen (Kurzweil, in [9]).

Our most powerful 21st century technologies—robots, genetic engineering, and nanotech—are threatening to make humans an endangered species [10].

Joy’s apocalyptic vision of the ‘grey-goo’ scenario—the horror of self-replicating nanorobots, destroying the world and leaving artificial grey goo after transforming all life on earth into nanosubstances—is reiterated in the best-selling novel ‘Prey’ (2002) by Michael Crichton. Crichton imagines the risks associated with creating tiny nanorobots, drawing on reference support to embellish his scenarios. Crichton’s novel is indicative of how debates about science are often made meaningful to the public through some form of literary narrative, or storytelling. To this end, Crichton’s contribution to how nanoscience has been imagined exemplifies what may be seen as the third pillar of science communication—science fiction—where science is the first, and science journalism is the second. Fiction often foregrounds the latest scientific discoveries, or points towards imminent realities, which society must consider, putting into the public domain the need for debate and dialogue. Science fiction—or fiction more generally—can be an effective way to shape the public imagination of new science and its challenge to humanity [11–13]. Indeed, Curtis [14] makes this point when discussing the role of documentary as an art form:

I find the best documentary reporting these days in things that don’t really classify as documentaries. Things like South Park, movies like The Big Short and American Honey, and the This Is England series. They are all about portraying the real world but they do it in ways that are surprising and imaginative. They make you look at things in new ways. Whereas traditional documentaries seem a bit stuck. I think this has happened because most of them have been moved off TV and into the art house cinema circuit. As a result they tend to play to what their audience already know—reinforcing their beliefs. Like the fact that bankers are bad. Or climate change threatens the world [14].

As for any new scientific discovery, the immediate applications of nanoscience have been considerably less radical than those that the advocates or critics had proposed [15]. Yet, the rise of knowledge about what nanoscience could do in the near future should not diminish the importance of speculations about where it could lead humanity in the long term. For instance, nanodots could create more efficient solar panels, which has revolutionary potential in terms of our energy usage. In this respect alone, there is an important opportunity for science communication to intervene through fiction, particularly as the science develops, to help people understand the likely timescales of applications. Indeed, there is no greater upstream location than that which is found before the metaphorical river has even begun to form, even if this requires accepting a certain level of uncertainty about the credibility of the speculation. While one may question whether science fiction constitutes engagement with science at all—since it may actually sacrifice science for drama—it would be catastrophic to the work of science communication to dismiss the importance of science fiction, especially when it is rooted within insights from cutting-edge research and development. After all, people within the science fiction creation community are often involved with the science industries, as the attendees of any science fiction convention reveals. Moreover, a precursor to interest in scientific news may be a wider interest in science fiction and future gazing.

Equally, aspirations for progress within science should not be separated from the peoples’ broader aspirations and anxieties for their lives, many of which are found within literature, rather than scientific journals. Thus, if one seeks to understand what kind of life is worth living, or what kind of society one seeks to bring about—as a basis for deciding which problems science should seek to resolve—then one must look beyond scientific possibility and, instead, formulate a deeper understanding of what progress in science affords for humanity. Thus, literature—whether it is classified as science fiction or not—can help us greatly in this pursuit. Such works as ‘*The Picture of Dorian Gray*’ inform humanity of the values that surround life extension or anti-ageing science, while the movie *Gattaca* [16] provides a glimpse into what life could be like in a world where non-therapeutic pre-implantation genetic selection decision-making exists. Alternatively, movies like *Her* (2014) assist our capacity to imagine what it would be like to build increasing complexity into our relationships with machines.

In the absence of sociological data, such speculations are often our best attempts to articulate what life may be like in a world where such technologies exist. A good example here is human cloning. Nobody knows how people would actually react to discovering that they have been cloned, or what kind of existential crises might arise, or what interpersonal relationships would be strengthened or weakened as a result. Yet, Caryl Churchill’s *A Number* (2006) does this precisely, in a poetic, beautiful, and sensitively staged two-person show. Alternatively, Orsen Welles’ *War of the Worlds* (1938) radio drama gives us a sense of what it might feel like to be invaded by aliens. Fiction has a role to play in helping develop our imaginations about what kinds of circumstances may come to fruition, if a certain course of action is taken. Moreover, society is reliant on the imaginations of great writers to provide compelling, detailed, intimate, and closely observed narratives about such futures, to assist us in imagining these possibilities. To judge the credibility of such work solely on the likely accuracy of the text is to miss one central consequence of its impact, which is that the text is a starting point for a deeper conversation about the future and how technology may change it.

The merit of utilizing literary works in the attempt to wrestle with questions about the future, even in science policymaking has some precedent historically. For instance, in the 2000s, when the US President’s Council on Bioethics [17] debated the consequences of the human genome project, such works as Anderson’s *The Nightingale* and Shelley’s *Frankenstein* were part of the Council’s reading list, made available publicly on their website. More widely, film-making has found its way into broader bioethics work, as in the case of the Nuffield Council on Bioethics’ film competition, which invited school children in the UK to make films explaining complex issues, or the Scottish Council on Bioethics film festival, which ran for ten years from 2010-2015. Literary works allow us to consider how scientific discovery imposes new narratives onto humanity—both burdens and opportunities. From Shelley’s *Frankenstein* to Huxley’s *Brave New World* to Atwood’s *Oryx and Crake*, the prospect of scientifically induced chaos and disorder lends itself to gripping narrative structures and a concern for the future. Such components are also crucial catalysts for generating interest in science for the wider public, especially as news space diminishes and entertainment space increases.

Equally, the shift in journalist practice over the last 30 or so years—from factual to emotional reporting—grows the importance of understanding the function of narrative in people’s lives, as a means of making sense of the world generally, and change in particular. Thus, it is little wonder that scientific organizations have invested more seriously into understanding the (social) science of communication. Indeed, this shift is also apparent within the field of bioethics, where the principal objective in communication terms is to articulate the implications of new scientific discoveries for how we live our lives. The detail of such explanations is to focus on things people care about, or, at least, how new biotechnological applications may disrupt the stories we tell about ourselves and our future. Furthermore, the rise of the bioethics essayist—such authors as Carl Elliott, Ben Goldacre, or Steve Fuller, who have, in different ways, become orators of humanity’s future—is indicative of how the communication turn in science has involved the expansion of futures expertise towards ethics [18].

These circumsrtances reval how literary texts become constitutive of the range of ways in which science becomes meaningful for a wide range of people [19]. Indeed, their powerful narratives are often the primary—or only—mechanisms through which science communication work reaches some audiences. Moreover, the separation between science journalism and science fiction is not clearly defined. Gorke and Ruhrmann [20] reveal how often science fact and science fiction converge in media reporting, but this should not lead us to conclude that publishing about such prospects constitutes more fake news. Indeed, one only has to look at the nature documentary format to appreciate that elements of fiction are critical to being able to tell stories. For example, it is impossible to capture sound of an elephant’s feet moving through a jungle and so sound is added afterwards, often created using instruments that most closely approximate the actual sound. Yet, this can create an impression of what the sounds is actually like, which is absolutely not true. Thus, whereas an elephant will move quite quietly through a forest, film-makers will create a sound effect that conveys impact and weight, which viewers then come to expect of this species. In this respect, science communication has always involved some elements of creative narrating and the crucial concern emerges when this is seen to seriously misrepresent what takes place. In any case, the relevant point here has to do with the role of fictionalizing science within science communication.

Yet, the concerns about how fiction is utilized in science story telling have been prominent for many years. Liakopoulos [21] considers that ‘the intense scientific debate that started in the early 1970s captured enough media attention to start an attempt of scientific popularization that sometimes verged on the border of science fiction’. (p. 8). Today, these established vehicles of science communication are augmented by a third dimension—the blogosphere—as the place where fiction and fact are blended through the blurred medium of digital space. The rise of Web 2.0 platforms, mostly described now as social media, brings with it additional challenges and opportunities to the communication of science, not least of which is the problem of fake news and, more precisely, distinguishing between material that is informative and that which misinforms.

## Nanopanic and the Persistence of Uncertainty

Despite the novelty of nanoscience, there are already hundreds of nanoproducts available for consumption, including food, packaging, clothes, paint, and electronic components. In this respect, it is perfectly possible for speculative science, which has considerable uncertainty and controversy surrounding it, to sit comfortably with the utilization of products that surround our lives. Indeed, this recognition that the presence of uncertainty does not hamper adoption is important to come to terms with, when considering how people make sense of new discoveries, products, or services. Yet, there remain many uncertainties about the risks associated with these products, especially with regard to engineered nanoparticles. For example, Song et al. [22] investigate the death of two female Chinese factory workers and the ill health of five others. The authors linked their death to nanoparticles found in fluid surrounding the lungs of the workers, outlining that the same nanoparticles were found in the substances these workers used [22]. Evidence of such concerns about nanoparticles has been apparent in various parts of the world. For instance, in recent years, activists in Grenoble protested the development of nanotechnology, scrawling ‘No Nano’ graffiti on the city’s bastille, perhaps articulating a degree of ‘moral panic’ [23] over how the development of science seems to take place without recourse to public opinion or adequate regulation. Laurent [24] provides a careful articulation of how such panics are brought about by the organized action of specific groups, who are then able to generate attention through their interventions.

This example also speaks to the complexity of fake news and why this term needs scrutiny. In the case of nanoparticles, one may assume that there is insufficient evidence to determine the risks associated with them entering our atmosphere. Furthermore, this uncertainty is sufficient to justify making a factual claim about the inadequacy of scientific control. Yet, this extension into concern about regulation transcend the original factual inquiry about risk , which then requires a different kind of expertise to justify. In this case, we observe how discourse about fake news obscures a wider concern about the assertion of power. The staging of these different moral perspectives on scientific development on some agora becomes integral to how the media communicate the issues around these scientific matters, and there are various political interests (formal and informal) that surround the communication of nanotechnology, each of which has different moral aspirations.

The dynamic nature of risk discourses explains why it is crucial to ensure that public engagement and science communication work is optimally placed to navigate this complex terrain. Indeed, over the years, nanotechnology has benefitted from some such initiatives. For instance, in July 2003, the Action Group on Erosion, Technology and Concentration (ETC Group) from Ottawa, Canada, released several papers on the potential risks of nanotechnology. The communiqué ‘Nanotech Un-gooed!’ suggested that the world is not yet ready for this latest and greatest technological revolution. The ETC Group argued that the likely negative effects upon the environment and health, along with the economic risks, require that we exercise caution in the development of nanoscience [25]. Moreover, they identified that extensive regulations and laws are urgently needed. The ETC emphasized the need for a wide public, scientific, and political debate, a common appeal among public engagement advocates. The subsequent question one may ask is what form this debate should have taken in the decade following these claims and, moreover, what would count as having undertaken adequate ‘public engagement’ to satisfactorily appease the critics who argue that there was not enough?

Yet, while a considerable amount of science communication and public engagement work on nanotechnology has taken place in the decade since—much of it informed by methodological insights, as nanotechnology research has matured—the need for communicating nanotechnology or engaging the public has not diminished. If anything, the need for public engagement with nanoscience has become even greater, particularly as more nanotechnology products become commercially available. Certainly, the nature of the questions that people face has shifted: from concerns about what humanity in general ought to do to questions of personal choice and morality over whether or not to embrace nanoproducts within our lives. In this respect, the need for public debate remains strong and unlikely to relent.

The open-ended nature of science communication has implications for how researchers, politicians, and scientists should regard such work—not as a means to an end, but as an end in itself. In short, there is no point at which either the institutions of science promotion or those who speak on behalf of the public—the media, politicians, public intellectuals—may conclude their communicating of scientific development. Instead, one may identify periods of prioritization in scientific communication, based on what are deemed to be the prominent, novel methods of any given period. In this respect, there is a need to think about science communication work as political work; there is no end point at which it will be complete, since the need for governance and the emergence of variation in human society demand a constant attention to such matters. Furthermore, there is no point at which the pursuit of science capital should cease, if we expect to remain beneficiaries of an informed and engaged population. Yet, there remain open questions on what are the best ways to engage the public on science. The next section examines the value of different models of public engagement, before considering the role of narrative within such work.

## Beyond Upstream Public Engagement

Over 20 years of public understanding work, three models of activity have become well known to science communicators: the deficit model, the dialogue model, and the upstream model (see [26]). The early years of science communication work focused on reducing the public deficit in knowledge about science. The research made a set of assumptions about what was required of scientists and what the public needed, which, today, are deemed lacking in understanding about what should be their ideal relationship. Yet, there still remain intimations of such commitments within much science communication work and so it is valuable to reiterate both their conceptual premises and their limitations. Thus, the first model of public understanding assumed a lack of public knowledge, a limited ability to communicate, and a lack of trust between science and its publics. To this end, public engagement work sought to address these issues, with the goal of promoting complicity in supporting the progress of science. Various researchers identified inadequacies with these assumptions, while also drawing attention to the inherent problem of a system whose goal was to pre-define how a public should regard scientific progress. It became apparent that utilizing scientific communication to build complicity was both unlikely to be effective and ideologically problematic. Moreover, the idea that public engagement should perform the role of educator, or that the public were ultimately ignorant, did not withstand sociological scrutiny.

Consequently, a second model of public understanding of science emphasized the need for dialogue between scientists and their publics [27]. This dialogical approach emphasizes bi-directionality and exchange between science and the public, providing space for interaction and negotiation of science’s goals and values. It also recognizes the importance of lay expertise as an informed knowledge, which plays an important role in constructing meanings around science through which one can construct and negotiate knowledge. This emphasis on active dialogue between scientists and society can be classified not only as the scientists’ engagement with citizens but also as engagement of citizens with science itself.

Examples of this form of public understanding research include influential studies of nanotechnology awareness within the USA, which draw on traditional social scientific methods. For example, key findings from a survey study among 1014 adults nationwide in the USA in August 2006 investigating public awareness about nanotechnology revealed that 42% of the respondents have heard nothing at all about nanotechnology, 27% had heard a little, 20% had heard some, with only 10% who had heard a lot, while 1% were not sure [28]. A similar study in 2005 also conducted for the Project on Emerging Nanotechnologies found that participants answering a pre-study questionnaire of 117 participants 54% outlined that they know almost nothing about the technology, 17% that they knew something, and 26% outlined that they knew a little ([29]: 8). Comparing these figures with a more recent study by PEN [30], it appears that little has changed. Hart [30] shows that, despite all communicative efforts, only 7% have heard a lot about nano, 17% heard some, and 26% heard a little about nanotechnology, in contrast to 49% who heard nothing at all and the remaining 1% being not sure. To this end, the studies demonstrated the need for further innovation in how public understanding work should take place.

Other findings from Hart [30] indicated that nearly 50% of the respondents felt unable make a statement about the nanotechnology’s benefits and risks. Among the rest, 20% think that benefits will outweigh the risks, while 7% believe that risks will outweigh the benefits, and 25% think that risks and benefits will be about equal (ibid). This showed the limited public awareness of discussions on benefits/risks and, by implication, the failure to fulfil the ethical aspiration of promoting informed understanding that would allow people to undertake autonomous decision-making around the use of nanotechnology within the society.

The shift in language from understanding to engagement reflects a third model of science communication, an era that has been characterized by power struggles between scientists and the public (and which may be the subject of the British Government’s concern within its Inquiry). Thus, one shortcoming of the dialogue model was its inability to address the power imbalance between scientists and lay audience. This has also led to what some have termed as a need for ‘upstream engagement’, where lay public are empowered to make influential decisions before science is funded and resourced [31]. On this basis, citizens are involved in debates and are given the chance to express their points of view. As a result, they can play a more active and influential role in decision-making about the future of science and technology. The Parliamentary Office of Science and Technology of the UK [32] articulated the premises of this model:

Dialogue is deliberative. Participants interact with experts, engage in the debate and.... are capable of forming their own opinions....The decisions encompass various points of view. Consideration is given to the stance of all those involved.

This way of thinking about science communication shows how actively engaging citizens involves more than just ‘participation’ in surveys and questionnaires, to public involvement with complex deliberate approaches and decision-making. These first examples of citizen engagement groups (as citizen juries) in the UK included *GM Nation?* in the years 2002 and 2003 and the *NanoJury UK* in 2005, which were followed—among others—by Nanodialogues—four experiments in upstream public engagement [33]. NanoJury UK was a two-way citizen’s jury on nanotechnology that ran in June and July 2005. It was organized by the University of Newcastle and Greenpeace and involved 16 members of the public, who formed the jury. After following an introduction on nanotechnology, they were further informed by a series of evidence sessions by expert witnesses. As a result of the investigation, the jury made 23 recommendations how to proceed, one of those, arguing for ‘more dialogue on new technologies and increased interactions between citizens and scientists’ ([31]: 125).

Nanodialogues (May 2005 to autumn 2006) were divided into four different experiments and was organized by Demos and the University of Lancaster. The experiments consisted of (1) a people’s inquiry on nanotechnology and the environment, (2) engaging research councils, (3) nanotechnology and development, and (4) corporate upstream engagement. All of the four experiments brought various stakeholders together, who were discussing different aspects of the technology as set agenda and scenarios for the individual experiment [33], As for NanoJury UK, participants of the first three experiments draw up recommendations, again prominently all of those recommendations included an urgent call for more ‘upstream’ public engagement work. Similarly, Burri [34] outlines an upstream engagement citizen panel model in Switzerland. Moreover, discussions in the literature describe the need for undertaking ‘consensus conferences’ to empower citizens, rather than just focus on influencing policy [35].

Despite these attempts to undertake more meaningful public engagement work, knowledge production, agenda-setting, decision- and policy-making processes, there remain limitations to this approach [36] and a number of explanations for this are apparent. First, there has not been enough good quality public engagement work on nanotechnology to allow it to enter mainstream awareness. To this end, science communicators might still be operating in the first or second mode of public engagement work, regardless of the third era approach being known, as evidenced by Lee and VanDyke [8]. Second, the work that has been undertaken on nanotechnology communication might be ineffective as a means of entering into the daily lives of people in a way that allows them to recall its significance. Finally, Wang [37] identifies the challenges connected with linking upstream engagement to relevant political decision-making in science. In this sense, the allure of upstream engagement might simply be an illusion and a more fundamental structural shift must occur in how science is organized through society to bring about more widespread engagement.

Yet, for the present purposes, the most remarkable finding is that the informants of the research in the USA [28–30] had difficulty recalling even the technical details of the science. To this end, one might conclude that expectations about the public’s ability to recall scientific detail—as an indicator of knowledge or understanding—should not be the primary focus of evaluating science communication. Rather, it may be more effective to focus on those aspects of the science that have implication for the people’s lives. Focusing on the moral and ethical implications of science, rather than its technical details, is a critical means through which to achieve such understanding. If people can be engaged on these issues more powerfully, rather than through just the scientific facts, then a more informed public can emerge. The evidence to support this claim draws on the earlier assumption that the most powerful way to engage people on technological transformations—in science fiction and so on—is through the moral issues they present. To this end, the remaining sections explore how narrative operates around understanding, how digital media provides a vehicle for such development, and, finally, how this combined approach nurtures what may be described as scientific citizenship.

### Narrative in Nanoethics

The millennial debates about genetics were characterized by attempts to do public engagement work *after* the research had been done. For nanotechnology, the expectation was somewhat different; it was a chance to engage the public before many of the applications had been developed. While the case is unproven as to whether early nanocommunication work brought about a more engaged or ethically empowered citizen, such aspirations resonate with the *upstream* model. Yet, while audiences might be more capable of screening for propagandist messages from transnational science organizations, there are major challenges that the development of nanotechnology implies for science communication generally and this is true also of the ethical challenges they generate.

First, there is a growing convergence of the sciences, which is creating new, hybrid ethical issues with which societies must grapple globally. This convergence has been evident for some years through the overlapping ground between bioethics, medical ethics, and environmental ethics. Each of these sub-disciplines emphasizes the importance of transdisciplinary approaches to research, drawing heavily on philosophy, law, sociology, and political science, for instance. Second, a corollary of these shifts is a growing sophistication in the bioethical method, which must be adopted within public understanding work. This approach has begun to encompass narrative studies, feminist approaches to bioethics, cultural theory, and aesthetics. Indeed, the emergence of ‘narrative ethics’ [38], as a way of making greater sense of the intricacies of ethical dilemmas, is indicative of this transition. For this reason, understanding how to harness moral issues through science communication work is a more complex challenge than was previously understood. While success in doing so may rely on traditional mechanisms of communication, such as literary cases, role play, case studies, and so on, there is now a greater appreciation for the complexity of these methods that should inform approaches to communication.

A final challenge to communicating nanotechnology is the proliferation of new kinds of ethically engaged community and new interest groups, which are changing the political landscape of the debate. Prominent examples of this include discussions about human enhancement, as medical technologies increasingly make possible the ability to make humans ‘better than well’ [39]. However, it also encompasses the ethics of life or health extension, which arises as a direct consequence of attending to age-related disease. Yet, research has shown that developing public engagement with the ethics of nanoscience may assist in promoting public understanding and awareness, each of which are essential components of informed decision-making. For example, the Eurobarometer 224 [40] emphasized the importance of ethics in scientific research. This emphasis is followed by a strong public demand that scientific research must take place within the boundaries of ethical and moral principles, outlining the need for a balanced assessment of prospects and risks of scientific research and progress. The breadth of this requirement may focus on the micro ethical decisions that take place at the point of application, or they may involve broad inquiries into assessing possible impacts on humanity, the environment and society, assessment of scientific research, as well as questions about the regulation of science within the policy authorities and scientific community. Yet, who should provide the expert commentary on what is ethically desirable for humanity, when faced with a range of scientific possibilities? This is a critical question in our times, as was made apparent in political debates within the UK and the USA over the 2016–2017 period.

When considering the evaluation and assessment of scientific research, as well as its moral and ethical implications, it is necessary to scrutinize what counts as *expertise* that will allow the discovery of an optimal relationship between the specialists and the broader public. As Turner [41] argues, media and scientific institutions use the lack and ambivalence of information to establish and expand expert power. Expertise and expert knowledge are highly influential facts in decision-making processes and the public’s understanding of scientific and ethical issues. Habermas [42] emphasizes this notion, claiming that expert culture makes democratic discussion impossible. Thus, expertise can control and manipulate the public, public perception, and attitudes towards science and scientific research, which effectively leads to disempowerment rather than empowerment.

While expertise can be valuable within debates about which science we should pursue, the right expertise is also needed when presenting the ethical dimensions of science and scientific research [6]. To this end, *scientific* expertise needs to be distinguished from *ethical* expertise within public understanding work, especially to avoid criticisms of media bias or conflict of interest. The power of experts is highly influential and reliance upon experts raises the question of public trust. In this context, understanding the effect of science communication involves taking into account how expertise is framed within communication systems. In turn, this involves understanding what processes elevate specific kinds of people to the position of experts and, hence, understanding how expertise assists—or detracts—from the public’s ability to comprehend any given science. This final point raises questions about whether the current mechanisms of constructing expertise about science—principally through the media—are adequate. If one concludes that it is not, then it is useful to look towards alternative communication channels for more effective forms of engagement.

### Social Media and Nanotechnology

As outlined earlier, traditional methods of science communication were informed by assumptions about a knowledge deficit within the public. To this end, research has focused on how the mass media might be better utilized to develop greater understanding. Yet, the mass media also has limitations, specifically its unidirectional logic. Until recently, television, print, and radio have relied on an editorial voice communicating to a consuming public, which has limited opportunity to respond. In recent times, this has begun to change through the rise of interactive content. For example, in the 1980s, talk radio became a boom industry and elements of this broadcasting remain prominent today. Television has progressively become bi-directional through the rise of chat shows and reality shows that involve the audience through existing telephonic systems that provide space for interaction. Indeed, this format has become so pervasive in television, that it has also generated disruptive interventions from producers. For example, consider the case of the Netherlands’ *Great Donor Show* (2007). In this case, the producers created a game show where the contestants were all in need of a kidney transplant. The winner of the show would receive the kidney being offered by a donor, and the audience was asked to vote on who should receive it. The world’s media reported widespread outrage about the show in advance of its broadcast, but, at the moment of concluding the show, it revealed itself as a hoax, an elaborate attempt to promote organ donation in a society where there is a shortage. The example shows how science, policy, and the public can intersect through an interactive communication platform.

Each of these formats espouses principles that are optimally expressed through digital communication systems, where the mode of participation involves high levels of interaction with content and where we see an attempt to replicate the social sphere in digital form. While the principle of interaction certainly preceded computer culture—consider the ‘Choose Your Own Adventure’ book series, for example, where readers choose the order in which they read the book based on questions they are asked within the pages—technology has taken interactivity a step further. Thus, the internet’s widespread utilization, via the release of the hypertext protocol, allowed people to explore hyperlinked documents in a network environment. This phase can also be referred to as Web 1.0, where the internet was publicly adopted into work and social life but still primarily being used to access information and resources being published on websites and hence limited to unidirectional ways and models of communication.

However, online communities can be distinguished from these other media in their development of a user-led editorial infrastructure. Unlike a newspaper, which might integrate audience-generated content via letters pages or even, today, blog comments from readers, the internet grows its editorial structure from the user community. To this end, digital environments present rich opportunities to progress public engagement upstream while encompassing multidirectional communication between various publics, stakeholders, and scientists. Indeed, new media platforms may be better suited than traditional media to building an engaged public, as their design takes more seriously the idea that public opinion can—and should—emerge from the bottom-up rather than be dictated from the top-down via editorial choices. Hence, online public engagement could advance the society’s participation in decision- and policy-making processes.

Outside of science, the internet has become fundamental to broader public discourses on critical social issues:

More than a billion people worldwide use the internet, both at work and in their social lives. Over the past three decades it has grown from an experimental research network and now underpins a range of new economic activities as well as activities and infrastructures that support our economies ([43]: 81).

With a daily user base of now over three billion people, and a range of new protocols and publishing opportunities, the Web 2.0 era—with its emphasis on *social experiences*—offers a new mode of connecting people through information. Social media allows more intelligent information retrieval, while allowing users to produce content and to collaborate and cooperate with each other, using powerful tools such as social networking sites, blogs, peer-to-peer file sharing and collaborative writing, and knowledge sharing tools such as wikis. This is not to say that it is an ideal system. After all, there is also a loss of authoritative perspectives that accompanies this fragmentation of information. Yet, Web 2.0 and its various networking and collaboration applications allow for public participation in a way that redefines the current boundaries of science communication It also makes it possible for the public to access expertise and knowledge, without being restricted by gatekeepers or media professionals. The web now serves as a platform where users have greater control over their messages and where more people can participate in the shaping of such conversations around information. As O’Reilly [44] outlined, Web 2.0 offers ‘...activation of collective intelligence and knowledge...’ and can thoroughly be defined as a great ‘solar system’ of collaboration and unique platform experience.

Participatory social media platforms offer ample opportunities for upstream public engagement in all aspects of science. Science blogs cover a wide range of issues and are creative, communication platforms for topics and issues, which hardly find their way into traditional media. Furthermore, the abundance of citizen science projects that now exist around the world is heavily reliant on social media and digital platforms more generally to create a network of common purpose. Yet, traditional media forms are also creating similar spaces through which scientists to reach their audiences, an increasing imperative in an era when newspaper readership is diminishing, and where many media outlets have gone into administration. Within blogs, authors and readers analyse, comment, articulate ideas, narrate, take up positions, discuss, and spread opinions. Blogs offer a coherent, comprehensive, and linked up way to report on science and scientific issues, to comment, and to classify.

No other communication medium allows such wide-ranging flexibility: articles can be written, modified, or extended immediately, with the results being visible and accessible within seconds. Whereas previously, traditionally scientific research results were published in specific journals, which were hardly accessible for the public, blogs allow authors to publish results and report on results directly, reducing the gap between scientist and public. The emergence of an open access era further calls for strategic thought on how best to make science meaningful when more scientific discoveries can be found by the general public. Moreover, the iterative journal article has emerged to replace the idea that a piece of work can be fixed or completely finished. In this respect, the scientific profession is also reconsidering its model for communicating knowledge in a way that more closely aligns with principles of social media publishing. Journal articles in the future may more closely resemble crowd sourced Wikipedia entries, rather than fixed, finished PDF documents, and this has huge implications for how humanity makes sense of its own history.

Social media environments abolish the asymmetry between experts and lay people and are providing space for counter-arguments and critique to influence debates. Indeed, social media is becoming a part of the emerging territory of citizen science, which is a crucial apparatus in the fledgling alliance between amateur scientists and professionals:

The Citizen Science Alliance is a collaboration of scientists, software developers and educators who collectively develop, manage and utilise internet-based citizen science projects in order to further science itself, and the public understanding of both science and of the scientific process. These projects use the time, abilities and energies of a distributed community of citizen scientists who are our collaborators (http://www.citizensciencealliance.org).

Feedback and responses are an integral part of science online; critique is no longer only happening in a closed academic scientific circle, but scientists and journalists are enabled to receive comments from the public and directly interact via commentaries, questions, and remarks with those, who are normally left behind: the public. Furthermore, from a professional background, blogs allow an interdisciplinary exchange, dialogue, and networking without disciplinary boundaries.

Participatory journalism or citizen/grassroot journalism adds another perspective to unidirectional mass media—the so-called ‘one to many’ model described by Shirkey [45]. Rushkoff [46] outlines that, due to increasingly centralized and profit-driven mainstream media, the ability to offer a multiplicity of perspectives is diminished. The provision of alternative and multiple perspectives on news and current events is established by user-driven online news websites and blogs [47]. As noted, blogs add further perspectives on news or report on issues, narrate stories using a personal perspective, allowing readers to gain insights and simply tell stories, which have been overlooked by or never gained entry into traditional media. Each of these elements provides a basis for developing a fourth model towards public engagement with science, focused on the idea of nurturing scientific citizenship.

## Conclusion: Towards a Theory of Scientific Agency

Each of the elements that I have discussed reveals the need for a fourth model of public understanding with science, which elevates the importance of nurturing scientific agency and which builds on the pursuit of developing ‘science capital’ (Archer et al. [3, 4]). These arguments require crucial consideration especially since habits around information consumption have changed in recent times. Today, more innovative methods are needed, which take into account the fact that people now read more news on their mobile devices than in newspapers. The collapse of expertise, diminished trust in the media through the growth of fake news, the rise of user generated content, and the emergence of citizen science all speak to the need for a much more sophisticated way of developing public engagement through science communication. Such work must attend to the shifting locations within which nurturing public understanding of science is possible, outside of formal, professional communication systems, towards informal, citizen-based environments.

As outlined by Bowman and Willis ([48], 13):

‘there is a new media ecosystem emerging where online communities discuss and extend the stories created by mainstream media. These communities produce participatory journalism, grassroot reporting, annotative reporting, and commentary’.

With easy opportunities to publish and distribute content via the internet, Bruns [47] argues that ‘no news organisation has the power anymore to choose what news is fit to print and what news is discarded and therefore the gates of publication have multiplied beyond all control ([47], 1)’.

First hand coverage within online news outlines the potential of citizens to observe and report more immediately than traditional media. The rise of alternative news sources and citizen journalism overcomes the significant shortcomings of traditional gatekeeper problems and offers a wider range of perspectives, opinions, and viewpoints by incorporating news beyond the views of politicians, leaders, and experts. As Bruns outlines ([47],13), ‘multiperspectival news is the bottom-up corrective for the mostly top-down perspectives of the traditional news media’. Furthermore, news coverage online is dialogic: it forms an ongoing conversation and exchange of opinions and views. In short, alternative media can easily fill gaps left by mainstream media and allow overlooked events or news to gain entry into public debate and agenda. We can observe the impact of these shifts by considering the case of nanotechnology.

Engaging with the ethics of science can be an effective way of promoting *engaged participation* around nanoscience and nanotechnology, because it provides an entry point into the debate for people regardless of education levels or awareness of the science. In this paper, engaged participation means, minimally, having contributed actively to discussions about such matters, either through posting responses online or to sharing information with others through social media. While so-called ‘clicktivism’ [49] can be to the detriment of a more consequential form of engagement with civil society—perhaps where people form beliefs or take decisions on the basis of the interaction—the expansion of media materials through user-generated content is a powerful indication of how the range of actors who are producing conversations about science has grown. Evidence to support this participatory mode of engagement is found in a range of publications about how political work is best accomplished online and offline, but it is also apparent within the ethos of citizen science. As Kolok et al. note, ‘active participation in the scientific process can lead to personal empowerment’ ([50], 626).

To this end, research into the communication of science generally and nanotechnology specifically should seek to harness the powerful device of moral narratives through which to activate public discourse. As a Nature editorial stated

Only by fully engaging at the outset with the cultural preconceptions of those audiences—by being what sociologists call ‘reflexive’—can science’s institutions do justice to their goal of engaging with citizens ([51], 451).

The consequences of developing scientific agency, rather than public understanding or public engagement as ends in themselves, are the achievement of personal investment into the development of science, not simply to its being understood, or even for that understanding to lead to informed decision-making or participation in the deliberative process. Rather, nurturing scientific agency speaks to a broader sense of investment, which corresponds with Arnason's notion of ‘scientific citizenship’, albeit without the irreconcilable “different intellectual roots” and “diverse positions and theoretical tensions” ([52], 938) it implies. Approaching public engagement with the intention of nurturing scientific citizenship and focusing on the ethical aspects of science, or as Perrault states, ‘keeping the focus on public values’ ([5], 111), provides a more sustainable approach than is achieved by the other models of science communication. Furthermore, it would more effectively ensure the ‘democratic involvement of citizens in science’ [53], which should be the primary consideration in the goals of science communication and public engagement.
